# On the inadequacy of nominal assortativity for assessing homophily in networks

**DOI:** 10.1038/s41598-023-48113-5

**Published:** 2023-11-29

**Authors:** Fariba Karimi, Marcos Oliveira

**Affiliations:** 1 Complexity Science Hub Vienna, 1080 Vienna, Austria; 2 Graz University of Technology, Graz, Austria; 3 Computer Science, University of Exeter, Exeter, UK

**Keywords:** Complex networks, Computational science

## Abstract

Nominal assortativity (or discrete assortativity) is widely used to characterize group mixing patterns and homophily in networks, enabling researchers to analyze how groups interact with one another. Here we demonstrate that the measure presents severe shortcomings when applied to networks with unequal group sizes and asymmetric mixing. We characterize these shortcomings analytically and use synthetic and empirical networks to show that nominal assortativity fails to account for group imbalance and asymmetric group interactions, thereby producing an inaccurate characterization of mixing patterns. We propose the adjusted nominal assortativity and show that this adjustment recovers the expected assortativity in networks with various level of mixing. Furthermore, we propose an analytical method to assess asymmetric mixing by estimating the tendency of inter- and intra-group connectivities. Finally, we discuss how this approach enables uncovering hidden mixing patterns in real-world networks.

## Introduction

Understanding how groups interact in networks is fundamental for uncovering mechanisms underlying diverse phenomena, from protein interactions to social communication^1–3^. Such group-level interactions often generate mixing patterns in networks, commonly assessed with single-valued measures such as nominal assortativity^4,5^. Though these measures help analyze group mixing concisely, they may be grounded on unrealistic assumptions about the network structure, which might produce imprecise estimates of group mixing tendencies, limiting our understanding of groups in networks.

Recent advances in relational data collection have enabled studies on mixing patterns to investigate fundamental processes that drive such tendencies^6^. In particular, much research has characterized homophily—nodes’ tendency to connect with alike—in a variety of social settings due to processes such as selective mixing and in-group favoritism^2,7^. For instance, sexual partnership networks are assortative by race in the United States, meaning that individuals form ties with partners of the same race more often than one would expect by chance^4^. Similarly, college students are more likely to have friendships with peers of the same gender, major, residence, and year^8^. These homophilic tendencies have been documented in various other social phenomena such as research collaboration^9^, artist partnerships^10^, lawmaking^11^, and book readership^12^. Beyond social networks, previous works have also demonstrated homophily in biological domains such as networks of protein similarity^13^ and dolphin companionship^14^.

Researchers in network science generally use the so-called nominal assortativity to characterize mixing patterns regarding categorical attributes (e.g., race, gender, protein type)^4^, such as those mentioned above. Nominal assortativity or attribute assortativity describes how intra- and inter-group connectivity diverges from what we expect solely due to degree connectivity of the nodes and groups. The advantage of this measure compared to other existing measures of homophily is that it takes into consideration connectivity patterns of groups to assess the statistical significance. Its straightforward definition produces an intuitive quantity that ranges from $-1$ (i.e., complete disassortative mixing) to 0 (i.e., neutral) to $+1$ (i.e., complete assortative mixing), which enables researchers to analyze group mixing in networks concisely. Recently, Cinelli and colleagues showed that the assortativity coefficient, *r*, is bounded, analogous to the constraints that exist on Pearson correlation coefficients^15^. They demonstrated that the assortativity coefficient can range between $r_{\text {min}}$ and $r_{\text {max}}$, which are dependent on the edge counts in the networks. This specifically implies that $r=1$ is only achievable when the sum of the inter-group edge counts is equal to the total number of edges in the network. Conversely, $r=-1$ is attainable solely in cases where this sum is zero. Crucially, this work exposes the influence of certain network attributes such as metadata assignment and degree sequence on the bounds of *r*.

**Figure 1:**
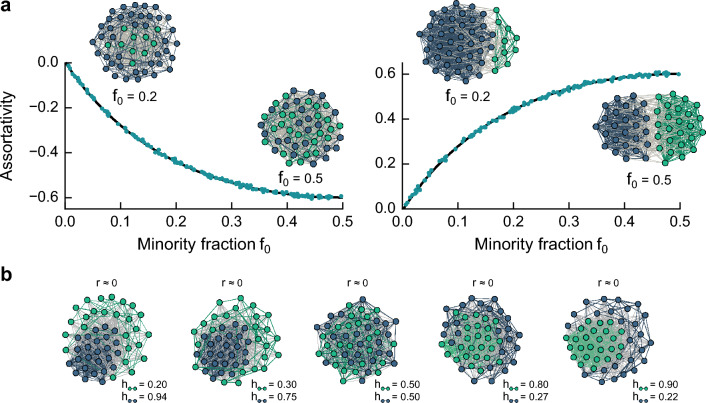
**Nominal assortativity misses relevant mixing patterns in networks.** **(a)** Nominal assortativity shows different mixing values for networks that have the same group mixing—a misrepresentation due to group-size imbalance. We generate these networks using a model with a group-mixing parameter *h* that corresponds to the probability of same-group nodes being connected; the generated networks are in a heterophilic regime with $h=0.2$ (left) and a homophilic regime with $h=0.8$ (right). These networks have a fixed group mixing *h* but varying minority fraction $f_0$. In the plots, solid lines represent the analytical formulation, whereas dots are values from simulations. **(b)** Nominal assortativity is a single-valued measure and ignores asymmetries in group mixing. In all scenarios, nominal assortativity is zero $r=0$, while there can be significant asymmetric mixing patterns between green and blue groups. In the plots, data points represent numerical simulation of networks with 300 nodes, repeated over 150 independent runs.

Here we demonstrate that nominal assortativity presents two fundamental inadequacies. First, it overlooks the group-size imbalance, implicitly assuming that groups are relatively equal. This assumption neglects that smaller groups have fewer possibilities to connect with themselves, misrepresenting mixing patterns in scenarios of group-size imbalance (see Fig. 1a). Second, the measure consists of a uni-dimensional value, only characterizing symmetric group mixing (or an average mixing). This restriction ignores potential asymmetries in networks, thereby missing relevant mixing patterns (Fig. 1b). Both inadequacies are problematic, particularly when analyzing real-world data and in the presence of minorities.

In real-world networks, groups tend to have unequal sizes, and some groups (i.e., minority groups) might be much smaller than the largest group. For instance, women are underrepresented in STEM fields, such as Computer Science and Physics, making them a minority group in professional networks^16–18^. When analyzing such networks and other imbalanced data sets, we must consider group sizes to estimate the likelihood of in-group mixing biases. Besides unequal group sizes, networks might display asymmetries in how groups interact. For example, in male-dominated scientific fields, established researchers could be primarily men due to historical first-mover advantages^18^. Thus, senior men have the resources to drive their collaboration network, implying that the tendency for male-male collaboration may not be the same as female-female collaboration^19,20^. In such settings, homophily is asymmetric, having different strengths for the minority and majority groups. These asymmetries, however, are lost when using a single-valued measure to characterize group mixing, such as nominal assortativity.

In this work, we demonstrate how nominal assortativity misses relevant mixing patterns in networks with unequal group sizes or asymmetric mixing and show how to tackle these shortcomings. First, we use generative network models with adjustable mixing parameters to show that nominal assortativity fails to recover the expected assortativity in synthetic networks. We characterize this limitation analytically and numerically by examining the relationship between assortativity, group size, and asymmetric mixing. Second, we propose the *adjusted nominal assortativity* and show that this adjustment recovers the expected assortativity from synthetic networks. Third, we propose to assess asymmetric mixing in networks by estimating group-mixing tendencies using our analytical formulations. Finally, we discuss how our approach enables characterizing hidden mixing patterns in real-world networks.

## Results

Nominal assortativity characterizes mixing patterns by assessing the significance of the intra-group. To that end, this definition employs the $B \times B$ mixing matrix ${\textbf {e}}$ to account for groups connectivity, where *B* is the number of groups, and each matrix element $e_{ij}$ corresponds to the fraction of edges connecting nodes from group *i* to nodes from group *j*. The nominal assortativity measure is then defined as follows:

1 $$\begin{aligned} r = \dfrac{\sum _i e_{ii} - \sum _{i} a_i b_i}{1-\sum _i a_i b_i}, \end{aligned}$$

where $a_{i}$ and $b_{i}$ are the fraction of edges that, respectively, begin and end at nodes from group *i*, defined as $a_{i} = \sum _{j} e_{ij}$ and $b_{i} = \sum _{j} e_{ji}$^4^. This definition produces an intuitive quantity that equals zero when groups lack intra- and inter-group tendencies (i.e., $e_{ii} = a_i b_i$). The quantity reaches to its maximum $r=1$ when intra-group ties dominate the network (i.e., $\sum _i e_{ii} = 1$) and becomes negative when inter-group ties are predominant.

### Nominal assortativity on networks with groups of unequal sizes

To examine how nominal assortativity represents mixing patterns, we use generative network models in which we have a prior knowledge on what to expect from the value of mixing. We aim to evaluate assortativity’s ability to recover the expected mixing value. More precisely, we generate random networks using a model with a tunable group mixing parameter *h* that corresponds to the probability of same-group nodes being connected, whereas its complement, $1-h$, is the probability of inter-group ties (see “Methods”). Here, we focus on the case of two groups, $B = 2$, in which nodes possess a binary attribute (e.g., red/blue, male/female). The case of beyond two groups is discussed in the Supplementary Note 4. We examine networks with a fixed *h* and varying group sizes, finding that nominal assortativity goes to zero as the minority group decreases in size (Fig. 1a). For example, when $h=0.8$ (i.e., homophily), assortativity can vary from 0.6 to 0, depending on the proportional size of the minority group, despite fixed group mixing.

To investigate why nominal assortativity varies with the minority fraction, we turn to the analytical formulation of the assortativity. Let us use a more general notion of group mixing in which $h_{ii}$ denotes the intrinsic tendency of a node from group *i* connecting to a node of the same group; its complement $h_{ij} = 1 - h_{ii}$ is the tendency of a node in group *i* to connect to a node in group *j*. Therefore, in a random network, the probability of finding an edge between group *i* and group *j* express as $p_{ij} = f_if_jh_{ij}$, where *f* corresponds to the proportional size of groups, implying that each mixing matrix element can be defined as $e_{ij} = p_{ij}/\sum _{ij} p_{ij}$, where the denominator is a normalizing factor.

Thus, $\sum _i e_{ii}$ and $\sum _i a_i b_i$ can be expressed as follows:

2 $$\begin{aligned} \sum _i e_{ii} = \dfrac{{f_0}^2 h_{00}+ {f_1}^2 h_{11}}{\sum _{ij} p_{ij}}, \end{aligned}$$

and

3 $$\begin{aligned} \sum _i a_i b_i = \dfrac{(f_0^2 h_{00} + f_0 f_1 h_{01} )^2 + (f_1^2 h_{11} + f_0 f_1 h_{10} )^2}{(\sum _{ij} p_{ij})^2}, \end{aligned}$$

where 0 and 1 are the labels for the minority and majority group, respectively. Finally, inserting Eqs. (2) and  (3) into Eq. (1), the nominal assortativity can be written as:

4 $$\begin{aligned} r = \dfrac{\dfrac{{f_0}^2 h_{00}+ {f_1}^2 h_{11}}{\sum _{ij} p_{ij}} - \dfrac{(f_0^2 h_{00} + f_0 f_1 h_{01} )^2 + (f_1^2 h_{11} + f_0 f_1 h_{10} )^2}{(\sum _{ij} p_{ij})^2}}{1-\dfrac{( f_0^2 h_{00} + f_0 f_1 h_{01} )^2 + ( f_1^2 h_{11} + f_0 f_1 h_{10})^2}{(\sum _{ij} p_{ij})^2}}. \end{aligned}$$

**Figure 2:**
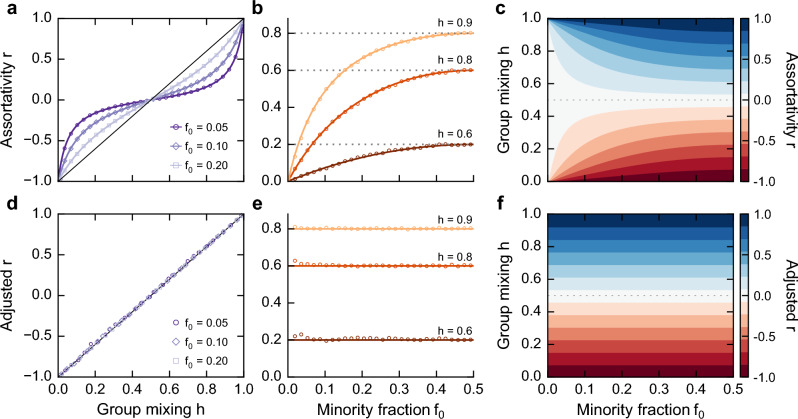
**Adjusted assortativity retrieves the expected assortativity in networks with group-size imbalance.** **(a)** Nominal assortativity has a group-size dependence that **(b)** underestimates the strength of group mixing in networks. **(c)** This underestimation is more severe in the presence of smaller minority groups. **(d)** We propose the *adjusted assortativity* that tackles this misrepresentation by adjusting for group sizes in the network. **(e)** The measure has a linear relationship with group mixing *h* and **(f)** is independent of group sizes. In all plots, solid lines represent the analytical formulation, whereas dots are values from simulations. The simulations are done on networks with 500 nodes and 60 independent runs.

This equation reveals that nominal assortativity is a function of group sizes $f_0$ and $f_1$. We verify this group-size dependency by comparing our analytical formulation with the assortativity measured on synthetic networks, finding a perfect agreement between Eq. (4) and simulations (Fig. 2a, b). Our results confirm the group-size dependence and reveal that this dependence increases with smaller minority groups (Fig. 2c). In contrast, when groups have similar sizes, we observe, as expected, a linear relationship between group mixing *h* and nominal assortativity. More precisely, when groups are equal in size, $f_0 = f_1 = 0.5$, Eq. (4) becomes $r = h_{00} + h_{11} - 1$. The group-size dependency occurs in other types of networks such as scale-free networks. For instance, we simulate the Barabási–Albert homophily (BA-Homophily) model, which incorporates group mixing preferences with the preferential attachment^21^, and demonstrate that nominal assortativity is a function of group sizes on such networks and in scenarios involving more than two groups (see Supplementary Notes 2 and 4). Overall, these findings imply that nominal assortativity is unadjusted for group sizes and introduces an artifactual bias into mixing analyses in imbalanced scenarios.

### The adjusted nominal assortativity

Here we propose to adjust the nominal assortativity for group sizes by normalizing the elements of the mixing matrix. This approach accurately retrieves the expected assortativity in networks, enabling us to assess mixing patterns in imbalanced networks. To that end, we define the *adjusted mixing matrix* $\textbf{e}^{\star }$, which accounts for the network’s pool of opportunities, namely, the fact that larger groups have more opportunities to connect. We define each element of the adjusted mixing matrix $\textbf{e}^{\star }$ to be

$$\begin{aligned} e^\star _{ij} = \dfrac{e_{ij}}{f_if_j}, \end{aligned}$$

where $f_k$ corresponds to the proportional size of group *k*. This adjustment ensures that the elements of the mixing matrix only represents the mixing tendencies (*h*) that are relevant for measuring intrinsic homophily and assortativity and not other factors. For instance, in the case of two groups, where original mixing elements are $e_{00} \simeq f_0^2 h_{00}$ and $e_{11} \simeq f_1^2 h_{11}$, the adjusted elements of the matrix are expressed as $e_{00}^\star \simeq h_{00}$ and $e_{11}^\star \simeq h_{11}$.

Moreover, we define the *adjusted nominal assortativity*, $r_{adj}$, as follows:

$$\begin{aligned} r_{adj} = \dfrac{\sum _i e^\star _{ii} - \sum _{i} a^\star _i b^\star _i}{1-\sum _i a^\star _i b^\star _i}, \end{aligned}$$

where $a^\star _{i} = \sum _{j} e^\star _{ij}$ and $b_{i} = \sum _{j} e^\star _{ji}$. This adjustment considers the effects of group-size imbalance on the mixing matrix, leading to a consistent assessment of mixing patterns in imbalanced scenarios.

We verify the proposed measure by generating synthetic data with different group-imbalance and mixing scenarios. We examine networks generated with a fixed *h* and varying group sizes, revealing that adjusted nominal assortativity accurately recovers the expected mixing independent of group sizes (Fig. 2d-e). Thus results show that the adjusted nominal assortativity has a linear relationship with group mixing *h*, regardless of $f_0$ and $f_1$ as expected (see Fig. 2f). We find similar results for scale-free networks and three-groups scenarios (see Supplementary Note 2 and 4). In sum, the adjusted nominal assortativity accounts for group sizes and pool of opportunities, enabling us to assess group mixing preferences accurately.

### Assessing group mixing in empirical networks

Next, we explore nominal assorativity in different real-world networks with unequal group sizes, showing that nominal assortativity underestimates the mixing patterns compared to the adjusted nominal assortativity (see Table 1). We analyze social networks of academic collaboration and face-to-face interactions with annotated binary gender information (see Supplementary Note 6 for detailed data descriptions)^22–24^. In most cases, assortativity *r* is lower than the adjusted assortativity $r_{adj}$, especially in the cases of small minority groups. For example, in the collaborative coding platform GitHub, where women are only 6% of the network, nominal assortativity is $r=0.04$, implying the absence of assortative collaboration; in contrast, the adjusted assortativity is $r_{adj}=0.16$, suggesting a potential gender assortativity. Similarly, nominal assortativity might mislead us to mistake changes in group mixing for changes in group sizes. For instance, in the collaboration network among computer scientists (DBLP), assortativity *r* increases from $r=0.04$ in 1980 to $r=0.10$ in 2010, which could imply a possible change in group mixing over time. However, this change might be merely due to the growth of the minority group. The minority size almost doubled, from 11% to 21%, and the adjusted assortativity indicates a stable mixing around $r_{adj} = 0.15$. Overall, these findings underscore the importance of accounting for group sizes when analyzing mixing patterns and the risks of ignoring group imbalance in networks.

**Table 1:** **Nominal assortativity and adjusted assortativity in empirical networks.**

Network	*N*	$f_0$	*E*	$E_{00}$	$E_{11}$	$E_{01}$	*r*	$r_{adj}$
APS (2000)	8285	0.11	9071	126	1539	7406	0.05	0.11
GitHub	311,755	0.06	1,537,570	7432	149,069	1,381,069	0.04	0.15
DBLP (2010)	170,984	0.21	322,052	17,468	91,738	212,846	0.10	0.14
DBLP (2000)	54,966	0.18	72,369	3123	18,101	51,145	0.11	0.16
DBLP (1990)	13,764	0.15	12,178	384	2701	9093	0.09	0.16
DBLP (1980)	2664	0.11	1765	24	274	1467	0.06	0.16
INFORMS (2010)	1426	0.16	1009	34	247	728	0.07	0.12
SocioPatterns 4	180	0.27	2220	182	762	1276	0.09	0.12
SocioPatterns 5	327	0.44	5818	1471	2348	1999	0.19	0.19

*N* denotes number of nodes, $f_0$ is the minority fraction, *E* is the total number of edges, and label 0 refers to the minority group and label 1 refers to the majority group.

### Mixing patterns in networks with asymmetric mixing

A single measure of assortativity reduces information about the $B\times B$ mixing matrix into a single value, leading to a concise measure but potentially missing relevant asymmetries in mixing. The idea that a single summary statistic may not be representative of a dataset is, of course, not new and has been shown in prior works^25^. More recently, Peel and colleagues showed heterogeneity in local assortativity^5^, and Piraveenan et al.^26^ showed the extent to which each node contributes to the measure of assortativity. Here, we pay a special attention to the asymmetric nature of group mixing while assuming no heterogeneity at the node level.

**Figure 3:**
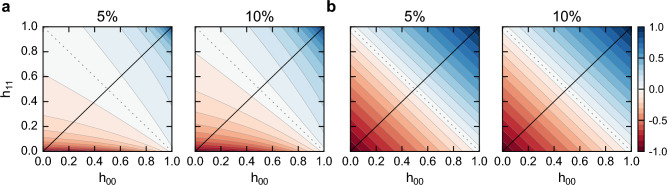
**Unidimensional measures of assortativity overlook asymmetry in networks.** (**a**) The nominal assortativity is dependent on group size in asymmetric cases, whereas (**b**) the adjusted version is size-independent. Yet both versions of assortativity ignore asymmetric mixing; they reduce a mixing matrix into a unidimensional value, producing a concise measure but missing asymmetry in networks. These measures might indicate an absence of mixing tendency despite significant asymmetric group mixing. In particular, both measures are zero when $h_{00} = 1 - h_{11}$ (i.e., the dashed lines). In the plots, each heatmap displays the respective measures in varying mixing scenarios of minority mixing $h_{00}$ and majority mixing $h_{11}$ in the presence of minority sizes $f_0=0.05$ and $f_0=0.10$.

To characterize *r* and $r_{adj}$ in asymmetric scenarios, we relax the assumption of $h_{00}=h_{11}=h$ and use our analytical formulation (Eq. 4) to evaluate the nominal assortativity over the whole parameter space of $h_{00}$ and $h_{11}$ (see Fig. 3). We find that the adjusted nominal assortativity is consistent and independent of group size in asymmetric cases, whereas the unadjusted version is size-dependent. Both measures, however, might characterize contrasting mixing patterns with the same value. In particular, these measures might indicate an absence of inter- or intra- group mixing tendency despite significant group mixing. For instance, when $h_{00} = 0.8$ and $h_{11} = 0.2$, the minority group has a strong homophilic tendency, whereas the majority has a strong heterophilic tendency; yet, nominal assortativity equals zero, incorrectly suggesting a lack of assortative or disassortative patterns (Fig. 3).

To better understand the underlying reason for this misrepresentation, note that when $r=0$, the numerator in Eq. (4) is zero, leading to the following equation:

$$\begin{aligned} \dfrac{{f_0}^2 h_{00}+ {f_1}^2 h_{11}}{\sum _{ij} p_{ij}} - \dfrac{(f_0^2 h_{00} + f_0 f_1 h_{01} )^2 + (f_1^2 h_{11} + f_0 f_1 h_{10} )^2}{(\sum _{ij} p_{ij})^2} = 0. \end{aligned}$$

Simplifying this equation by using the expression of Eq. (6), we find that $h_{00} = 1 - h_{11}$ satisfies this condition. In other words, in many cases when nominal assortavitiy reports a zero value (i.e., lack of any dis/assortative preferences), the group mixing tendencies could be widely different. These findings show that compressing the mixing matrix into a single value, such as assortativity, can hide relevant asymmetric mixing patterns that are present in networks. It is worth noting that paying attention to asymmetries in mixing patterns between groups is important in other applications, such as the emergence of core-periphery structures^27^.

### Assessing asymmetric mixing patterns in networks

In order to assess asymmetric mixing among groups, we propose to turn to the mixing probabilities in a network given an assumption of its generative process. For example, in a random homophilic networks described earlier (ER-Homophily), the diagonal of the mixing matrix can be expressed as:

$$\begin{aligned} e_{00} = \frac{{f_0}^2 h_{00}}{\sum _{ij} p_{ij}} \quad \text {and}\quad e_{11} = \frac{{f_1}^2 h_{11}}{\sum _{ij} p_{ij}}, \end{aligned}$$

which can be re-written as follows:

5 $$\begin{aligned} h_{00} = \frac{E_{00}}{E}\frac{\sum _{ij} p_{ij}}{{f_0}^2} \quad \text {and}\quad h_{11} = \frac{E_{11}}{E}\frac{\sum _{ij} p_{ij}}{{f_1}^2}, \end{aligned}$$

where

6 $$\begin{aligned} \sum _{ij} p_{ij} = {f_0}^2 h_{00} + {f_1}^2 h_{11} + {f_0}{f_1}h_{01} + {f_0}{f_1}h_{10}, \end{aligned}$$

and *E* is the total number of edges, and $E_{00}$ and $E_{11}$ are the number of intra-group edges of the minority and majority groups, and $e_{00}$ and $e_{11}$ are fraction of intra-group edges normalized by *E*. By combining the equations above, $\sum _{ij} p_{ij}$ can be expressed as:

7 $$\begin{aligned} \sum _{ij} p_{ij} = \frac{2 f_0 f_1}{1-e_{00}(1-f_1/f_0) - e_{11}(1-f_0/f_1)}. \end{aligned}$$

By using Eqs. (5) and  (7), we can retrieve $h_{00}$ and $h_{11}$ from data, given we know basic information about the network (i.e., *E*, $E_{00}$, $E_{11}$, and $f_0$).

We verify this method by generating networks with varying mixing parameters and compare the estimated parameters with the ground truth in Supplementary Note 5. Similar methodology can be applied to scale-free networks, finding equivalent results (see Supplementary Note 6). Though this approach requires prior knowledge about the underlying generative processes in networks, it is plausible to argue that many small-scale and large-scale social networks often fall into these two categories of topologically random or scale-free structure^28–30^. Once the plausible topology is identified by examining the degree distribution, the appropriate formulation can be used to extract the group mixing asymmetries. In Supplementary Note 3, we discuss the relationship between asymmetrical homophily and adjusted nominal assortativity in undirected networks.

## Discussion

Despite its popularity and relative accuracy in capturing homophily and assortative mixing in a variety of networks, nominal assortativity can produce distorted assessments of mixing patterns in networks with unequal groups and asymmetric mixing. In this work, we demonstrated this inadequacy and proposed ways to tackle these limitations.  By using generative network models with adjustable mixing, we show that nominal assortativity fails to assess homophily accurately in certain scenarios. Our results demonstrate (1) the need for accounting for group sizes in such analyses and (2) the inability of single-valued measures to capture asymmetries in networks.

To tackle these limitations, we develop two approaches to assess group mixing in networks. First, we propose adjusted nominal assortativity to solve the group-size limitation, which accurately recovers the expected symmetric assortativity from networks. Our analysis of real-world networks reveals that nominal assortativity underestimates the strength of mixing patterns compared to the adjusted assortativity. Second, we propose to assess asymmetric mixing in networks by estimating the intra-group mixing probabilities accounting for group size differences and other group-level topological features. It is worth mentioning that there are a variety of other segregation and assortativity measurements in the social network literature beyond the nominal assortativity. Future works should focus on comparing the sensitivity, equivalency, and compatibility of those measurements against each other and baseline scenarios similar to this paper and the previous efforts^31^.

Accurately measuring biases in group mixing in social networks is crucial because mixing biases affect perception of minorities^32^, access to social capital^33^, and algorithmic visibility^34^, to name a few. Our work lays a novel foundation by proposing an accurate measure of assortativity that can be applied to a wide range of social networks. Better assessment of group-level tendencies and asymmetries in networks provides the means to understand how diverse groups interact—a fundamental step for uncovering mechanisms governing our social lives.

## Methods

### Random networks with group mixing

To analyze assortativity in networks, we develop a simple model that incorporates group mixing and random tie formation in networks. In this model, an edge between two nodes depends on their group memberships via a stochastic process by tuning the homophily parameter ranging from 0 to 1, $h \in {0,1}$. That means the probability of a node from group *i* to establish a tie with a node from group *j* is denoted as $h_{ij}$. The probability of connecting with nodes of the same group is thus the complementary function so that $h_{ii} = 1 - h_{ij}$, likewise $h_{ji} = 1 - h_{jj}$. At each simulation step, one random node is selected, and it connects to a random target based on this probability. Note that this model has equivalencies with a simple version of stochastic block models in cases where group memberships are known and are the drivers of block formations^35^. Analytical derivations of the mixing probabilities are described in Supplementary Note 1, and the code is available in the GitHub repository.

### Supplementary Information

Supplementary Information.

## Data Availability

The sources of all empirical data used in our analyses are described in Supplementary Note 6.

## Code availability

All relevant code used in this study will be available at https://github.com/macoj/assortativity.
