# Supplementary material for: On the inadequacy of nominal assortativity for assessing homophily in networks
Source: Sci Rep. 2023 Nov 29;13:21053. doi: 10.1038/s41598-023-48113-5 (PMC10686992; doi:10.1038/s41598-023-48113-5)
Supplement: Supplementary file 1 — Supplementary Information. [file 41598_2023_48113_MOESM1_ESM.pdf]

On the inadequacy of nominal assortativity for assessing homophily in networks

Supplementary Material

F. Karimi[1] and M. Oliveira[2]

1 Complexity Science Hub Vienna, 1080 Vienna, Austria
2 Computer Science, University of Exeter, Exeter, United Kingdom

Supplementary Note 1: Analytical derivation of assortativity in ER-Homophily

ER-homophily stands for Erdős–Rényi random-homophily network model. Let f_i denotes fraction of nodes of group i and f_j fraction of nodes of group j. For example, if in a network 20% of nodes are minority i and the rest and the majority j, $f_i = 0.2$ and $f_j = 0.8$ respectively. Let us consider the elements of the mixing matrix. In a random network, the probability of two nodes of the same attribute to be connected depends on their proportion and homophily:

$$p_{ii} = {f_i}^2 h_{ii} \text{ and } p_{jj} = {f_j}^2 h_{jj}. \tag{1}$$

Similarly, the probability of two nodes of different attributes to be connected depends on (a) the probability of i attaches to j, $p_{ij} = f_i f_j h_{ij}$ or (b) the probability of j attaches to i: $p_{ji} = f_j f_i h_{ji}$. Thus, the total link probabilities sum to:

$$\sum_{ij} p_{ij} = p_{ii} + p_{ij} + p_{ji} + p_{jj} \tag{2}$$

Note that for observed undirected networks it is not possible to distinguish p_{ij} and p_{ji} and the elements of the mixing matrix is the sum of these two probabilities: $e_{01} = e_{10} = p_{ij} + p_{ji}$. Given these probabilities, one can write the elements of the mixing matrix for categorical attributes as follows:

$$e_{ii} = \frac{{f_i}^2 h_{ii}}{\sum_{ij} p_{ij}} \text{ and } e_{jj} = \frac{{f_j}^2 h_{jj}}{\sum_{ij} p_{ij}} \tag{3}$$

Supplementary Note 2: Nominal assortativity in BA-homophily networks

Here we extend the analysis to networks with scale-free degree distributions. In this case, we use the BA-Homophily network model [1] (stands for Barabási-Albert) as a natural extension of the ER-Homophily model. In the BA-Homophily network model, the mechanisms for tie formations are homophily and preferential attachment. In this case, we tune the group mixing parameter h, as we did before, and examine the differences between the nominal assortativity and homophily. Fig. 1 shows the relation between homophily value from the synthetic model and nominal assortativity. As in the case of random networks, nominal assortativity deviates from linear function with group mixing when minority size is relatively small (Fig. 1 first row). It is interesting to note that this deviation is not symmetrical compared to the one in the random network due to the interplay between preferential attachment and homophily.

Comparing adjusted assortativity (Fig. 1 second row) for random networks and scale-free networks reveals a meaningful difference. Even after adjusting for group size effects, homophily and adjusted assortativity do not correspond completely. This effect is the inherent part of the preferential attachment mechanism embedded in the BA-Homophily model. According to this model, the combination of pair-wise homophily and a node's degree determine link formation. Thus, accounting for the group size effect is not enough to recover the expected homophily from the assortativity measure.

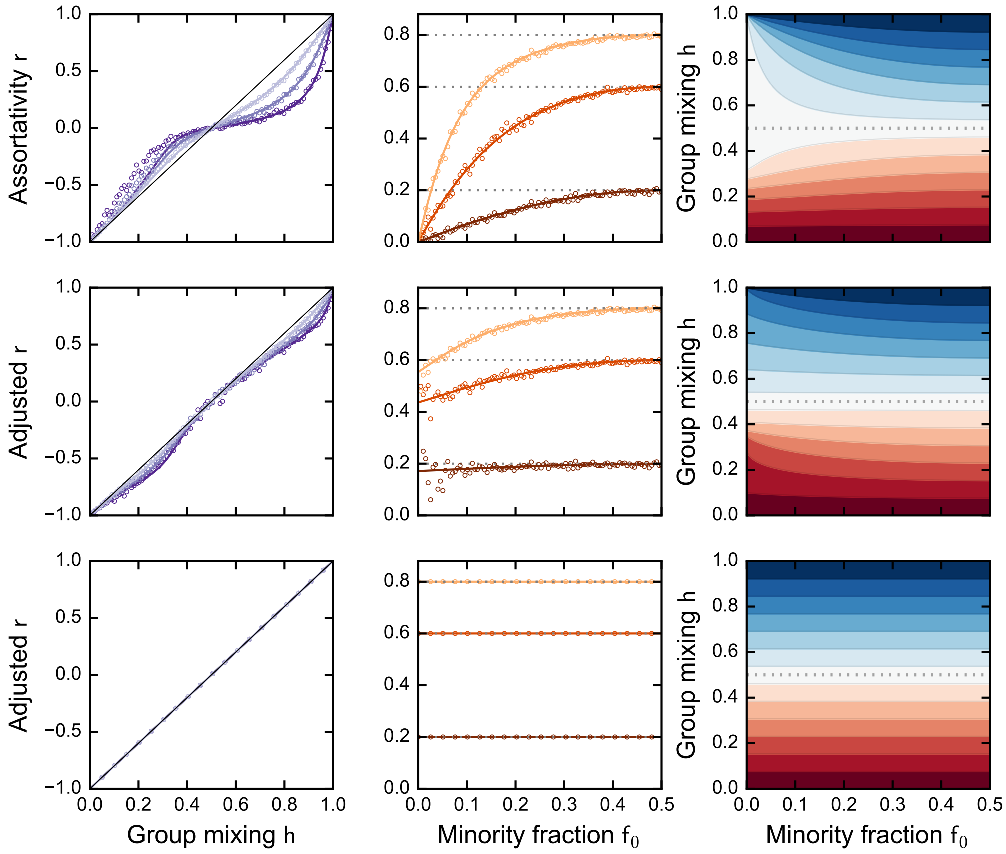

Fig. 1: The relationship between assortativity, homophily, and minority size in scale-free homophilic networks. First row displays how assortativity values changes as a function of homophily (group mixing) and minority size. We expect that group mixing should have a perfect linear relationship with assortativity. Second row displays the relationship between the adjusted assortativity and homophily. Third row displayes degree-corrected adjusted assortativity. The dots are numerical values while the solid lines are analytical values.

Here, we provide analytical derivations for estimating homophily in BA-homophily networks [1]. In a network with E edges, let us call E_{ii} the number of edges linking two nodes of the group i (in-group links), and similarly E_{jj} the number of edges linking nodes of the group j. The proportion of in-group links, $e_{ii} = E_{ii}/E$, is given by:

$$e_{ii} = \frac{f_i^2 h_{ii}(1-\beta_j)}{f_i^2 h_{ii}(1-\beta_j) + f_i f_j h_{ij}(1-\beta_i) + f_i f_j h_{ji}(1-\beta_j) + f_j^2 h_{jj}(1-\beta_i)}. \tag{4}$$

Similarly for the group j:

$$e_{jj} = \frac{f_j^2 h_{jj}(1-\beta_i)}{f_i^2 h_{ii}(1-\beta_j) + f_i f_j h_{ij}(1-\beta_i) + f_i f_j h_{ji}(1-\beta_j) + f_j^2 h_{jj}(1-\beta_i)}, \tag{5}$$

where β_i, β_j are the exponent of the degree growth for group i and j. These exponents can be calculated analytically (see [1]) or numerically by using maximum-likelihood estimate. Note that the exponent of the degree growth has an inverse relationship with the degree exponent (γ) as follows:

$$\gamma = -(1/\beta + 1). \tag{6}$$

Thus, by estimating the exponent of the degree distribution in empirical networks, one can estimate β. Once the degree growths are known, h_{ii} and h_{jj} can be calculated from system of equations Eq. (4) and Eq. (5). By rearranging the equations, we find:

$$h_{ii} = \frac{E_{ii}}{E} \frac{\sum_{ij} p_{ij}}{{f_i}^2(1-\beta_j)} \text{ and } h_{jj} = \frac{E_{jj}}{E} \frac{\sum_{ij} p_{ij}}{{f_j}^2(1-\beta_i)}, \tag{7}$$

where

$$\sum_{ij} p_{ij} = f_i^2 h_{ii}(1-\beta_j) + f_i f_j h_{ij}(1-\beta_i) + f_i f_j h_{ji}(1-\beta_j) + f_j^2 h_{jj}(1-\beta_i). \tag{8}$$

and $\sum_{ij} p_{ij}$ can be rewritten as:

$$\sum_{ij} p_{ij} = \frac{f_i f_j (2-\beta_i-\beta_j)}{1 - e_{ii}(1 - f_j/(f_i(1-\beta_j))) - e_{jj}(1 - f_i/(f_j(1-\beta_i)))}. \tag{9}$$

We highlight the similarities and differences between Eq. (7) and Eq. (5) in the main text. The mixing values for scale-free networks are not only dependent on the size of the groups (f^2) but also the degree exponent of the groups ($1-\beta$). In this case, adjusted assortativity that corrects for the group size balance may not be enough to account for other structural properties such as asymmetries in group's degree. In this case, adjusted assortativity could be calculated by correcting both group size balance and degree exponent. To account for group size imbalance as well as group's degree growth differences (β) we define each element of the adjusted mixing matrix $\mathbf{e}^\star$ to be

$$e^\star_{ij} = \frac{e_{ij}}{f_i f_j (1-\beta_i)}. \tag{10}$$

Therefore, the degree-corrected adjusted nominal assortativity is expressed as follows:

$$r_{adj}^{deg-corrected} = \frac{\sum_i e^\star_{ii} - \sum_i a^\star_i b^\star_i}{1 - \sum_i a^\star_i b^\star_i}, \tag{11}$$

where $a^\star_i = \sum_j e^\star_{ij}$ and $b_i = \sum_j e^\star_{ji}$. Fig. 2 shows the impact of this adjustment in different scenarios of mixing.

Note that this estimation requires prior knowledge about the generative processes in the corresponding network. While it worth reflecting on this point, the correction by the group size demonstrates sufficient improvement in estimating the actual value of group mixing. We recommend considering the degree-corrected adjustment in social networks that demonstrate scale-free distributions with extreme values of homophily and group imbalance.

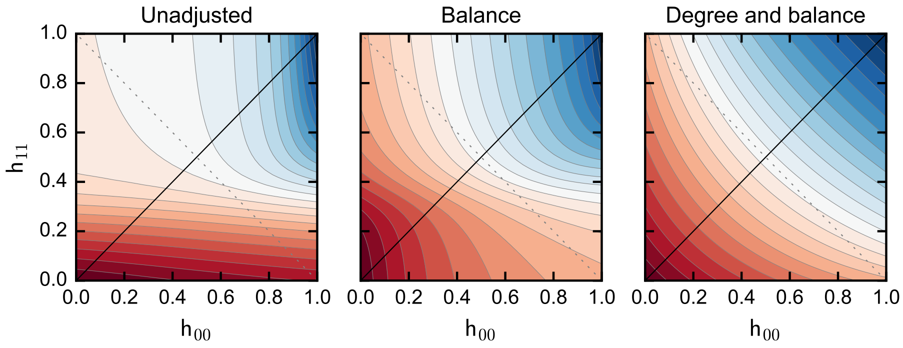

Fig. 2: Assortativity and asymmetric mixing in scale-free networks. (**Left**) Nominal assortativity, (**middle**) Adjusted assortativity, (**Right**) Degree-corrected adjusted assortativity, for minority size of $f_0 = 0.1$.

Supplementary Note 3: On the directionality of the links

It is worth noting that whether a network is directed or undirected would affect the assortativity in scenarios with asymmetrical homophily. Essentially, when networks are directed, e_{ij} is distinguishable from e_{ji} in the mixing matrix, while for undirected networks, the off-diagonal of the mixing matrix are identical. Let us consider the adjusted mixing matrix where groups size is corrected and each element of the matrix demonstrates the homophily mixing $\mathbf{e}^\star$ to be

$$e^\star_{ij} = h_{ij}, e^\star_{ji} = h_{ji}.$$

In such conditions, the adjusted nominal assortativity r_{adj} can be re-rwitten as follows:

$$r_{adj} = \frac{\sum_i e^\star_{ii} - \sum_i a^\star_i b^\star_i}{1 - \sum_i a^\star_i b^\star_i} = \frac{0.5(h_{ii} + h_{jj} - 1)}{1 - 0.5},$$

where

$$r_{adj} = h_{ii} + h_{jj} - 1.$$

As it can be seen from this derivation, recovering the directionality in the mixing matrix result in a linear relationship between nominal assortativity and two values of homophily. In undirected networks, this relationship becomes sub-linear. Also note that under the assumption of symmetric and equivalent homophily for two groups, we recover $r_{adj} = 2h - 1$, the linear relationship that we have observed in previous figures.

Supplementary Note 4: Assortativity in networks with more than two groups

To demonstrate that the problem of unequal group sizes affects the assortativity measure even when there are more than two groups, we construct networks comprised of three groups and assess the coefficient under varying conditions of group imbalance and mixing. We use the ER-Homophily model to generate these networks and label the groups as 0, 1, and 2. We assign Group 2 with a constant fraction size, denoted as f_2, and incrementally change the fraction size of Group 1 from 0 to $1 - f_2$ in our simulations; consequently, we distribute the remaining nodes to Group 0, following the equation $f_0 = 1 - f_1 - f_2$.

Next, by fixing a value for h when generating networks, we evaluate how variations in group size fractions affect the assortativity measure. Figures 3, 4, 5, and 6 illustrate scenarios where f_2 is equal to 0.6, 0.7, 0.8, and 0.9, respectively. Our analysis reveals that the unadjusted assortativity is dependent on f_1. For comparative purposes, we juxtapose this with the balanced scenario where all group fraction sizes are equal (i.e., $f_0 = f_1 = f_2 = 1/3$), represented by the dashed line in the figures. Our findings show that the adjusted assortativity mirrors the balanced case, thus confirming that the adjusted assortativity remains unaffected by the imbalance in group sizes.

We also explore the relationship between h and both adjusted and unadjusted assortativity in the context of three-group scenarios. Once again, we use the ER-Homophily model to generate networks that have constant group sizes f_0, f_1, and f_2 while we vary the mixing value h withing the interval $[0, 1]$. We measure the assortativity of these networks and compare them with a balanced baseline, where all groups are of equal size, as shown in Fig. 7.

We find that the unadjusted assortativity deviates from the baseline, whereas the adjusted assortativity mirrors the behavior of the baseline. We note that the lower limit of assortativity is higher than -1.0, which corroborates the assertion in Newman's seminal paper [2] that the theoretical minimum assortativity can be determined using the following equation:

$$r_{\text{min}} = \frac{\sum_i a_i b_i}{1 - \sum_i a_i b_i}, \tag{12}$$

which is also graphically represented in Fig. 7. In conclusion, the adjusted assortativity mimics the balanced case, allowing comparisons of the coefficient even when group sizes vary.

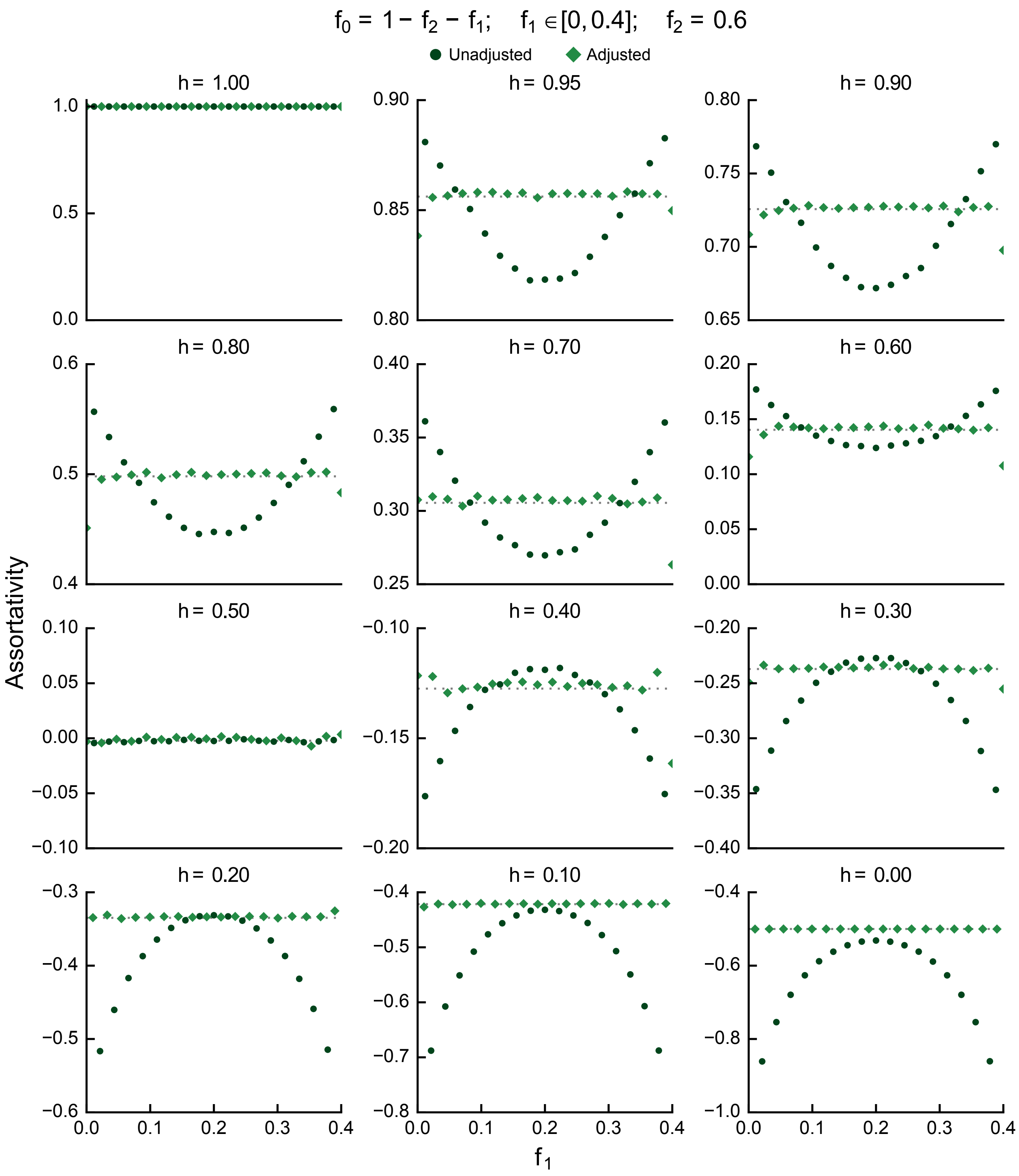

Fig. 3: Nominal assortativity shows different mixing values for networks that have the same group mixing also in the case of three groups ($f_2 = 0.6$). In each plot, we generate networks with different group sizes but same group mixing h, finding that the unadjusted assortativity depends on group sizes. In contrast, the adjusted assortativity is unaffected by group sizes, similar to the balanced case. The dashed line depicts a balanced scenario where all group fraction sizes are equal (i.e., $f_0 = f_1 = f_2 = 1/3$). The fraction size of Group 1 varies from 0 to 0.4.

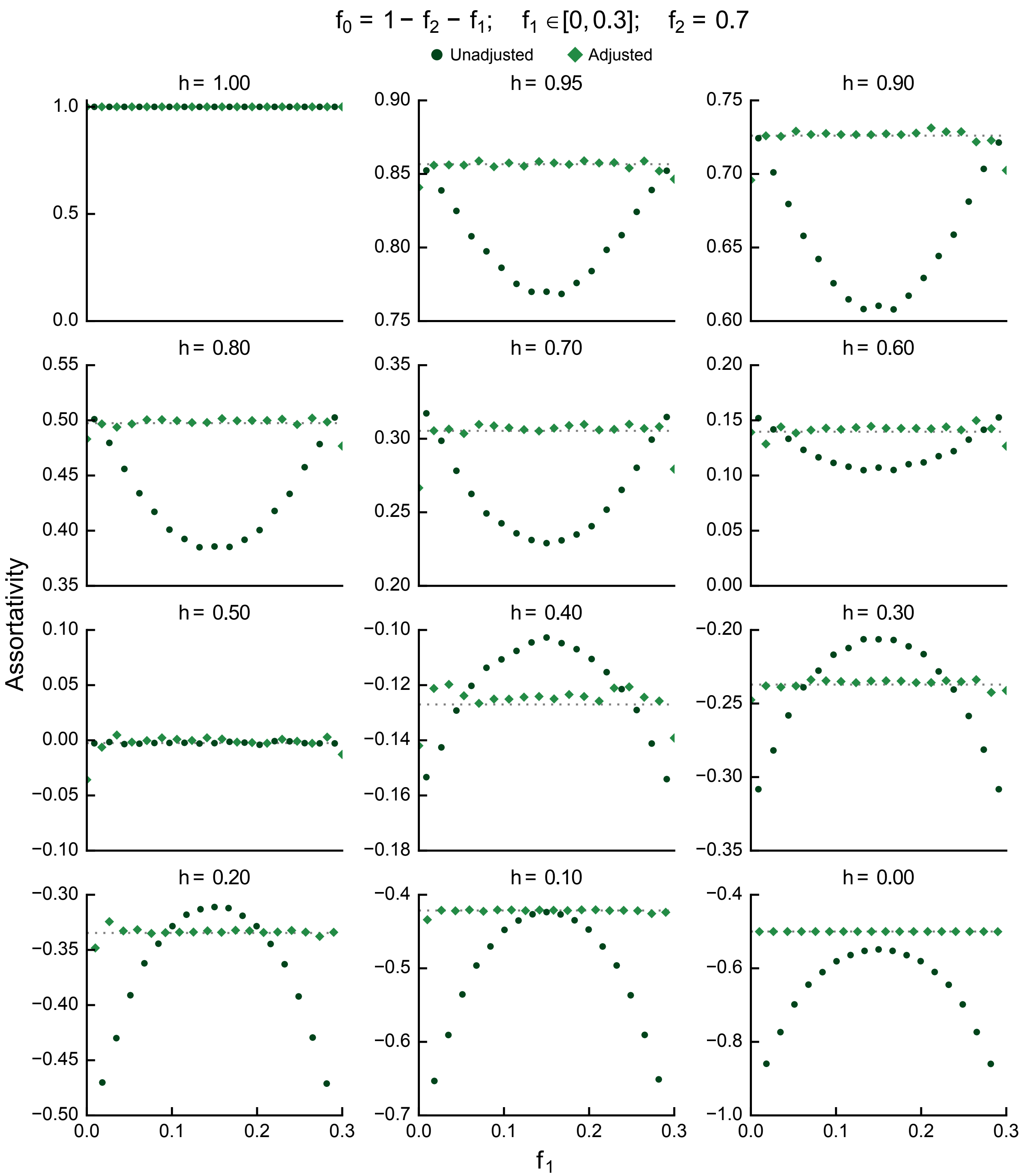

Fig. 4: Nominal assortativity shows different mixing values for networks that have the same group mixing also in the case of three groups ($f_2 = 0.7$). In each plot, we generate networks with different group sizes but same group mixing h, finding that the unadjusted assortativity depends on group sizes. In contrast, the adjusted assortativity is unaffected by group sizes, similar to the balanced case. The dashed line depicts a balanced scenario where all group fraction sizes are equal (i.e., $f_0 = f_1 = f_2 = 1/3$). The fraction size of Group 1 varies from 0 to 0.3.

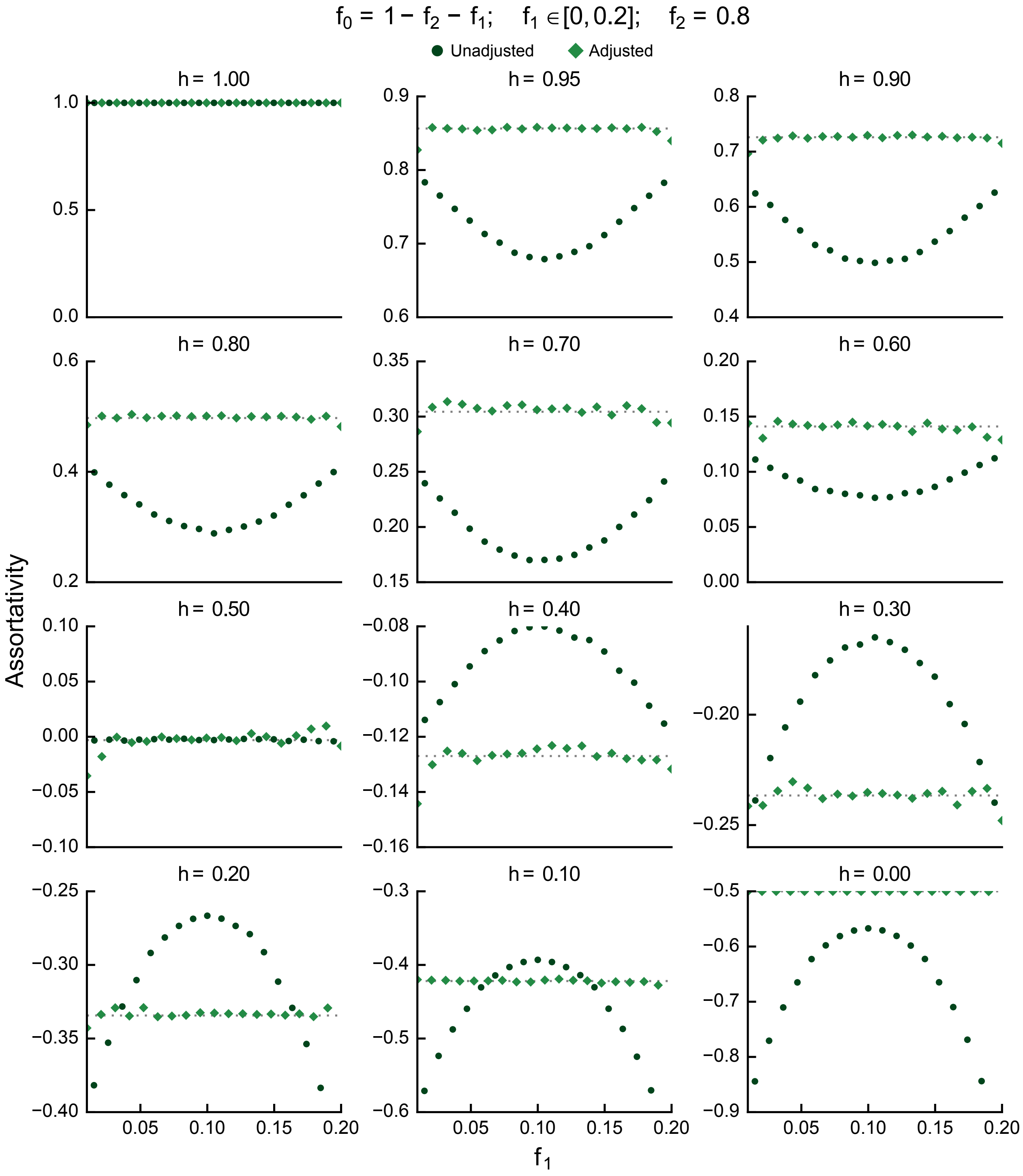

Fig. 5: Nominal assortativity shows different mixing values for networks that have the same group mixing also in the case of three groups ($f_2 = 0.8$). In each plot, we generate networks with different group sizes but same group mixing h, finding that the unadjusted assortativity depends on group sizes. In contrast, the adjusted assortativity is unaffected by group sizes, similar to the balanced case. The dashed line depicts a balanced scenario where all group fraction sizes are equal (i.e., $f_0 = f_1 = f_2 = 1/3$). The fraction size of Group 1 varies from 0 to 0.2.

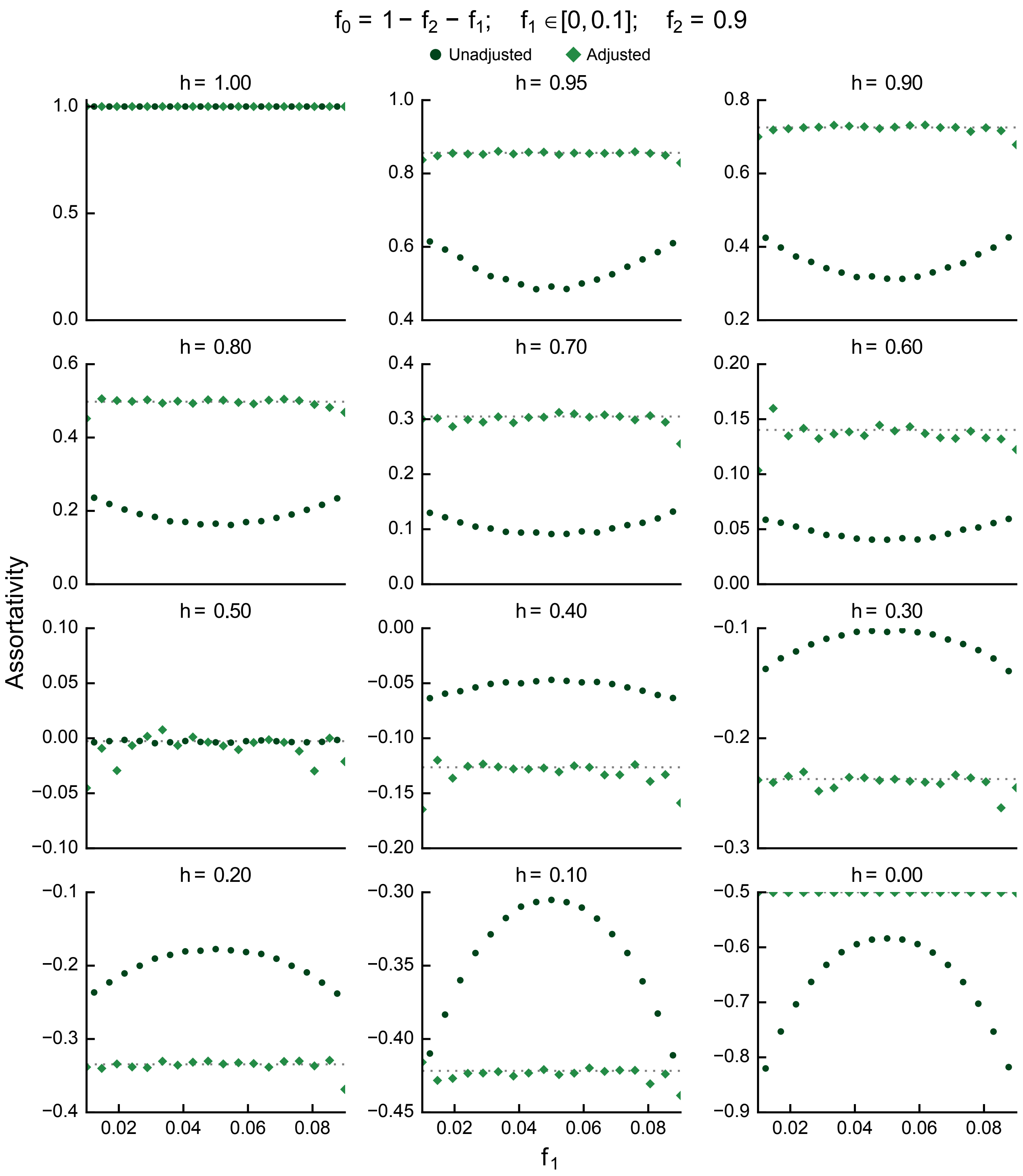

Fig. 6: Nominal assortativity shows different mixing values for networks that have the same group mixing also in the case of three groups ($f_2 = 0.9$). In each plot, we generate networks with different group sizes but same group mixing h, finding that the unadjusted assortativity depends on group sizes. In contrast, the adjusted assortativity is unaffected by group sizes, similar to the balanced case. The dashed line depicts a balanced scenario where all group fraction sizes are equal (i.e., $f_0 = f_1 = f_2 = 1/3$). The fraction size of Group 1 varies from 0 to 0.1.

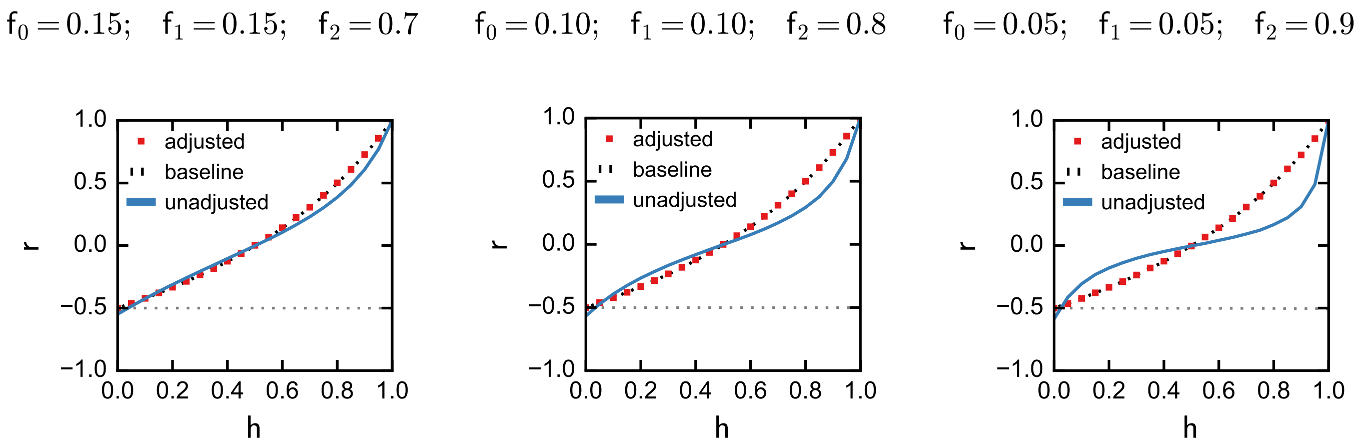

Fig. 7: Adjusted assortativity is unaffected by group size imbalance. In each plot, we generate networks using the ER-Homophily model, keeping constant group sizes, while the mixing value h varies within $[0, 1]$. The curves showcase that unadjusted assortativity deviates from the balanced scenario, while adjusted assortativity replicates the behavior of the balanced scenario. This illustrates that adjusted assortativity allows comparisons even when groups differ in size. We note that the lower bound of assortativity is higher than -1.0, as posited in Newman's paper [2], defined by Eq. (12), and depicted with a dashed horizontal line.

Supplementary Note 5: Measuring asymmetric mixing patterns in networks

In our analysis of asymmetric mixing within homophilic networks, we focus on two network models: ER-Homophily (random networks) and BA-Homophily (scale-free networks). In the case of the ER-Homophily model, we analytically determine group mixing $\mathbf{h}$ using Eq. (5) and Eq. (7) from the main paper, which we rewrite here for convenience:

$$h_{00} = \frac{E_{00}}{E}\frac{\sum_{ij} p_{ij}}{{f_0}^2}, \quad h_{11} = \frac{E_{11}}{E}\frac{\sum_{ij} p_{ij}}{{f_1}^2}, \quad \text{and} \quad \sum_{ij} p_{ij} = \frac{2 f_0 f_1}{1 - e_{00}(1 - f_1/f_0) - e_{11}(1 - f_0/f_1)}.$$

By knowing the group sizes and inter- and intra-group links (i.e., E_{00} and E_{11}), we can compute h_{00} and h_{11}. To illustrate this approach, we generate 200 networks, each containing 500 nodes, using h_{00} and h_{11} values values that were randomly selected from a uniform distribution. We applied this method with f_0 set to values of 0.1, 0.2, 0.3, 0.4, and 0.5. Our results confirm that our approach accurately determines the mixing in these synthetic networks. Fig. 8 shows a perfect agreement between our analytical calculations for h_{00} and h_{11} and the ground truth, which corresponds to the group mixing values used in the network generation process.

To assess asymmetric mixing in scale-free networks (BA-Homophily model), one might consider using Eq. (7) and Eq. (9) coupled with the estimation of the degree growth exponents (i.e., β_0 and β_1). However, estimating the degree growth exponents poses challenges, potentially leading to imprecise estimates of group mixing. A more reliable approach involves using the analytical formulation of the exponents to find the corresponding values for h_{00} and h_{11} using an optimization algorithm. Here we use the analytical formulation of β_0 and β_1 as described in [1]. Precisely, we use an optimizer to find the h_{00} and h_{11} values that best matches the inter- and intra-group links (i.e., e_{00} and e_{11}) and the β_0 and β_1 initially estimated from data, while having both h_{00}, h_{11}, β_0, and β_1 as the parameters of the optimization.

We evaluate this approach by generating networks with different levels of mixing and comparing the estimates with the ground truth. We generate 200 networks, each containing 1000 nodes, using h_{00} and h_{11} values randomly drawn from a uniform distribution. We set f_0 equals to 0.1, 0.2, 0.3, 0.4, and 0.5. Fig. 9 demonstrates the accuracy of the estimated values of h_{ii} and h_{jj} in comparison to the ground truth values for various minority sizes in the scale-free homophily model. While our method consistently provides accurate estimates for h_{11}, regardless of minority size f_0, the estimates for h_{00} can diverge from the ground truth in cases involving small minority size.

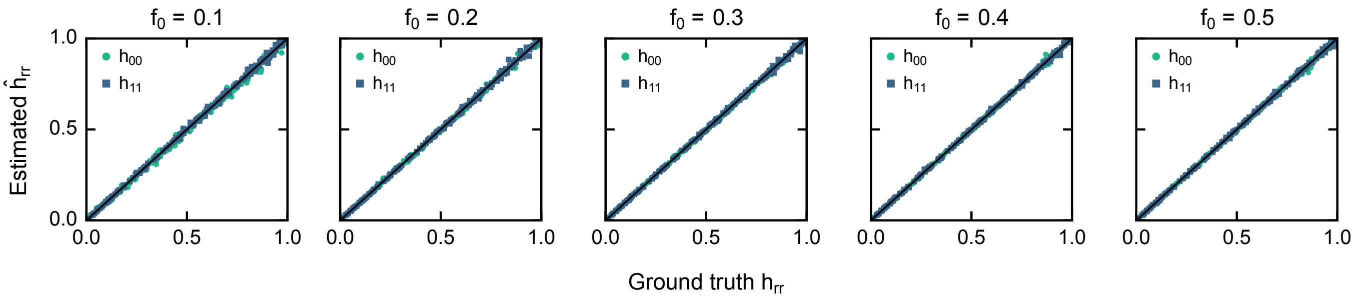

Fig. 8: Our approach recovers group mixing in synthetic networks generated with the ER-Homophily model. In each plot, we generate networks using distinct pairs of group mixing h_{00} and h_{11}, and then we employ our analytical formulation to estimate these values from the data. Our results show that this approach accurately assesses asymmetric group mixing in networks. The analytical estimations of the homophily values (y-axis) are in agreement with what we expect from the ground truth values (x-axis). Each plot corresponds to a different minority fraction f_0. Within these plots, h_{00} and h_{11} are the values of group mixing for the minority and majority groups, respectively.

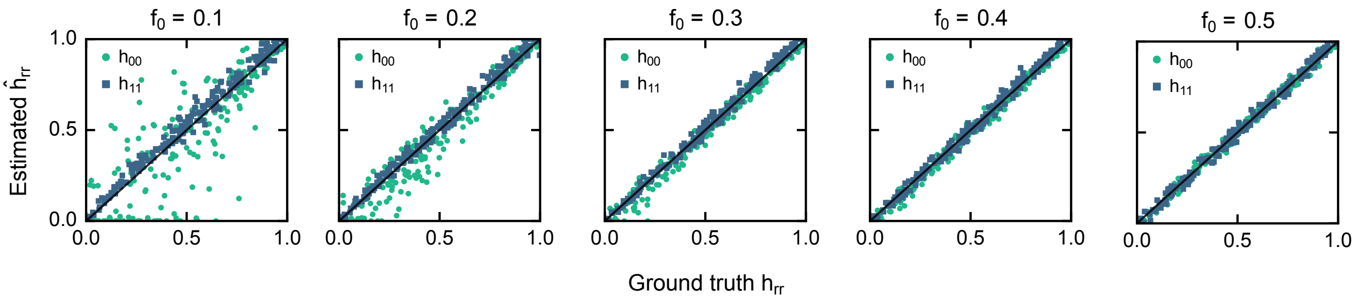

Fig. 9: Our approach recovers group mixing in synthetic networks generated with the BA-Homophily model. In each plot, we generate networks using distinct pairs of group mixing h_{00} and h_{11}, and then we the analytical formulation of β_0 and β_1 as described in [1] to find h_{00} and h_{11} values from the data. Generally, the estimations of the homophily values (y-axis) are in agreement with what we expect from the ground truth values (x-axis). However, while our method consistently provides accurate estimates for h_{11}, regardless of minority size f_0, the estimates for h_{00} can diverge from the ground truth in cases involving small minorities $f_0 < 0.2$.

Supplementary Note 6: Data sets

APS. American Physical Society dataset (APS) consists of all publications and citations records that was published in one of the sister journals of the APS platform. Gender identification was done based on our proposed method of combining first-name approach together with image-based approach by retrieving relevant images based on search query of first-name + family-name [3; 4]. More detail of this data can be found in [5].

Github. Github is a collaborative programming environment. The network is one snapshot of the community in which nodes are programmers and links are follower-followee relationships. Each programmer has a self-identified information including the first name and family name. We used similar gender inference methods described above to infer the gender of the programmers.

DBLP. DBLP is a computer science bibliography database that contains publication records of computer scientists over the coarse of 50 years. We used the gender inference methods described above to infer gender of the researchers in this database. The data and methods can be found in [6; 7].

INFORMS. These data contain the nodes and edges of a bipartite authorship network constructed from publications in 16 peer-reviewed journals affiliated with the INFORMS society from 1952 to 2016. Publication records were acquired using INFORMS PubsOnline (in the form of BibTeX entries) and the CrossRef REST API. The gender annotation was already done by the authors using the commercial package Genderize.io API with high accuracy threshold. The data and methods can be found in [8].

SocioPatterns 4 and 5. SocioPatterns 4, contains the temporal network of face-to-face contacts between students in a high school in Marseilles, France. The first dataset gives the contacts of the students of three classes during 4 days in Dec. 2011, and the second corresponds to the contacts of the students of 5 classes during 7 days (from a Monday to the Tuesday of the following week) in Nov. 2012. More information about the data and the network can be found in [9]. SocioPatterns 5, corresponds to the contacts and friendship relations between students in a high school in Marseilles, France, in December 2013, as measured through several techniques. More information about the data and the network can be found in [10].

Supplementary Note 7: Nominal assortativity for the case of equal groups

Let us assume that h_{00} denotes the intrinsic tendency of a node from group 0 connecting to a node of the same group; its complement $h_{01} = 1 - h_{00}$ is the tendency of a node in group 0 to connect to a node in group 1. Therefore, in a random

network, the probability of finding an edge between group 0 and group 1 express as $p_{01} = f_0 f_1 h_{01}$, where f corresponds to the proportional size of groups, implying that each mixing matrix element can be defined as $e_{01} = p_{01}/\sum_{01} p_{01}$, where the denominator is a normalizing factor. Therefore, $\sum_i e_{ii}$ and $\sum_i a_i b_i$ for two groups can be expressed as follows:

$$\sum_i e_{ii} = \frac{{f_0}^2 h_{00} + {f_1}^2 h_{11}}{\sum_{ij} p_{ij}},$$

and

$$\sum_i a_i b_i = \frac{(f_0^2 h_{00} + f_0 f_1 h_{01})^2 + (f_1^2 h_{11} + f_0 f_1 h_{10})^2}{(\sum_{ij} p_{ij})^2},$$

where 0 and 1 are the labels for the minority and majority group, respectively. When groups are equal in size, $f_0 = f_1 = 0.5$, we can simplify the assortativity r. First, $\sum_{ij} p_{ij}$ can be rewritten as:

$$\sum_{ij} p_{ij} = {f_0}^2 h_{00} + {f_1}^2 h_{11} + f_0 f_1 h_{01} + f_0 f_1 h_{10} = (0.5)^2 (h_{00} + h_{01} + h_{11} + h_{10}) = 2(0.5)^2.$$

We note that $h_{00} + h_{01} = 1$ and $h_{11} + h_{10} = 1$, so

$$\sum_i e_{ii} = \frac{{f_0}^2}{\sum_{ij} p_{ij}} (h_{00} + h_{11}) = (0.5)^2 (h_{00} + h_{11})/(2(0.5)^2) = (h_{00} + h_{11})/2$$

and

$$\sum_i a_i b_i = \frac{(f_0^2 h_{00} + f_0 f_1 h_{01})^2 + (f_1^2 h_{11} + f_0 f_1 h_{10})^2}{(\sum_{ij} p_{ij})^2} = \frac{(0.5)^4 (h_{00} + h_{11}) + (0.5)^4 (h_{11} + h_{10})}{4(0.5)^4} = 1/2.$$

Finally, we insert these values back to the equation r, and we get:

$$r = \frac{\sum_i e_{ii} - \sum_i a_i b_i}{1 - \sum_i a_i b_i} = \frac{(h_{00} + h_{11})/2 - 1/2}{1 - 1/2} = h_{00} + h_{11} - 1.$$

References

[1] Karimi, F., Génois, M., Wagner, C., Singer, P. & Strohmaier, M. Homophily influences ranking of minorities in social networks. *Scientific reports* **8**, 11077 (2018).

[2] Newman, M. E. J. Mixing patterns in networks. *Physical Review E* **67**, 026126 (2003).

[3] Karimi, F., Wagner, C., Lemmerich, F., Jadidi, M. & Strohmaier, M. Inferring gender from names on the web: A comparative evaluation of gender detection methods. In *Proceedings of the 25th International conference companion on World Wide Web*, 53–54 (2016).

[4] Karimi, F. & Vujovic, S. image-gender-inference. `https://github.com/gesiscss/image-gender-inference` (2017).

[5] Kong, H., Martin-Gutierrez, S. & Karimi, F. Influence of the first-mover advantage on the gender disparities in physics citations. *Communications Physics* **5**, 1–11 (2022).

[6] Jadidi, M., Karimi, F., Lietz, H. & Wagner, C. Gender disparities in science? dropout, productivity, collaborations and success of male and female computer scientists. *Advances in Complex Systems* **21**, 1750011 (2018).

[7] Karimi, F., Jadidi, M., Wagner, C. & Lietz, H. Collaborations of computer scientists between 1970 and 2016 .

[8] Bravo-Hermsdorff, G. *et al.* Gender and collaboration patterns in a temporal scientific authorship network. *Applied Network Science* **4**, 1–17 (2019).

[9] Fournet, J. & Barrat, A. Contact Patterns among High School Students. *PLoS ONE* **9**, e107878 (2014).

[10] Mastrandrea, R., Fournet, J. & Barrat, A. Contact patterns in a high school: A comparison between data collected using wearable sensors, contact diaries and friendship surveys. *PLoS ONE* **10**, e0136497 (2015).
